# Switching to transdiagnostic treatment of worry and rumination following poor early response to CBT-E

**DOI:** 10.1186/2050-2974-2-S1-O9

**Published:** 2014-11-24

**Authors:** Bronwyn Raykos, Emma Dove, Sharon Ridley, Anthea Fursland

**Affiliations:** 1 Centre for Clinical Interventions, Perth, Australia

## 

We describe the treatment of a patient with BN (plus comorbid major depression and generalised anxiety disorder) who completed stage one and stage two of CBT-E at a specialist eating disorders outpatient clinic. Despite good compliance with all components of the treatment, the patient did not achieve an early rapid reduction in binge-eating and purging by the stage two progress review and was considered to be at-risk of suboptimal treatment outcome. Her repetitive negative thinking (worry and rumination) was identified as significantly interfering with progress, and the primary trigger for episodes of binge-eating and vomiting. In light of this, it was decided to evaluate the usefulness of abandoning CBT-E and switching to metacognitive therapy (MCT) for repetitive negative thinking (RNT). RNT is defined as cognitive perseveration on negative themes. MCT has been shown to demonstrate excellent outcomes in patients with anxiety and depressive disorders at the same clinic. Switching to a transdiagnostic meta-cognitive was associated with achieving optimal outcomes at the end of treatment. It is recommended that clinicians routinely assess the degree to which patients have achieved an early response to CBT-E and systematically evaluate the effectiveness of switching or pursuing CBT-E in this patient group.

This abstract was presented in the **Treatment in Community and Inpatient Settings** stream of the 2014 ANZAED Conference.

